# Nucleophilic substitution reactions of monofunctional nucleophilic reagents with cyclotriphosphazenes containing 2,2-dioxybiphenyl units *Dedicated to precious supervisor Prof. Dr. Adem Kılıç on his retirement


**DOI:** 10.3906/kim-1907-45

**Published:** 2020-02-11

**Authors:** Esra TANRIVERDİ EÇİK, Hanife İBİŞOĞLU, Gönül YENİLMEZ ÇİFTÇİ, Gizem DEMİR, Eda ERDEMİR, Fatma YÜKSEL

**Affiliations:** 1 Department of Chemistry, Faculty of Science, Atatürk University, Erzurum Turkey; 2 Department of Chemistry, Faculty of Science, Gebze Technical University, Gebze, Kocaeli Turkey

**Keywords:** Cyclotriphosphazene, 2,2′ -dihydroxybiphenyl, NMR, X-ray

## Abstract

The nucleophilic substitution reactions of mono- and bis-spiro-2,2′ -dioxybiphenyl cyclotriphosphazenes (3 and 4) with cyclopropanemethylamine (5) and aniline (6) were performed in the presence of trimethylamine in THF. Five novel cyclopropanemethylamino- and anilino-substituted spiro-2,2′ -dioxybiphenyl cyclotriphosphazene derivatives (7–11) were obtained from these reactions. The molecular structures of the new cyclotriphosphazene derivatives (7–11) were characterized by elemental analysis, MALDI-TOF MS, FT-IR, and NMR ( ^31^ P and ^1^ H) spectroscopies. The structure of the spiro-(2,2′ -dioxybiphenyl)-bis-(anilino)-cyclotriphosphazene (11) was also determined by single-crystal X-ray crystallography.

## 1. Introduction

Among the inorganic heterocyclic rings, cyclotriphosphazenes have attracted interest due to their use as scaffolds for the preparation of a wide variety of functional and speciality materials [1–5]. The cyclotriphosphazene core is renowned for the extreme robustness of its phosphorus-nitrogen backbone and 6 very active phosphorouschlorine bonds, which enable nucleophilic substitution reactions with mono and difunctional reagents [6–10]. The substitution type in the cyclotriphosphazene ring is directed by electronic, steric, and mechanistic effects [10–13]. When electron donating groups are on the N _3_ P _3_ ring, the positive charge at the phosphor atoms reduce and monofunctional alcohols react almost exclusively by the nongeminal pathway [14]. Reaction of the cyclotriphosphazene core with secondary amines result in predominantly nongeminal substitution, whereas primary amines, such as t-butylamine and aniline, prefer geminal disubstitution due to steric or polar reasons [10,11,13,15]. The geminal pathway is attributed to a rate-determining ionization step prior to attack by the nucleophile (S _N_ 1), while associative substitution (S _N_ 2) is attributed to nongeminal isomeric product formation [10,13] .

Other advantages of the cyclotriphosphazene scaffold are the possibility to control the reaction pathways in substitution reactions and obtain new derivatives that have different properties depending on the characteristics of the functional groups [3,4,9,10]. The combination of the cyclotriphosphazene core with one or more chromophores or specific side units has received increasing attention because of their applications in medicinal agents, organic light-emitting diodes and fluorescence probes [3,5,6,16–20]. Spirocyclic phosphazene derivatives containing 2,2′ -dioxybiphenyl units play a role in building metallic architectures and the design of fluorescence chemosensors [7,13,21 –25]. Generally, different functional moieties containing nitrogen atoms are attached to spirocyclic cyclotriphosphazenes for the preparation of multiside coordination ligands and coordination polymers [7,22,25]. Although nucleophilic substitution reactions of the mono- and bis-spiro-2,2′ -dioxybiphenyl cyclotriphosphazenes with various reagents, such as pyrazole, pyridine, pyrene, benzoxazine, and o-vanillin have been investigated in detail [1,7,18,21,24], to the best of our knowledge, there have been no reports about cyclopropanemethylamino- and anilino-substituted spiro-2,2′ -dioxybiphenyl cyclotriphosphazene derivatives thus far. Due to the role of the cyclotriphosphazene ring in the design of new molecules and the advantages of functional groups containing nitrogen atoms, this study focused on the reactions of monoand bis-spiro-2,2′ -dioxybiphenyl cyclotriphosphazenes with new reagents, such as cyclopropanemethylamine and aniline. The newly cyclopropanemethylamino- and anilino-substituted spiro-2,2′ -dioxybiphenyl cyclotriphosphazene derivatives (7–11) were designed and synthesized (Scheme). The molecular structures of the newly synthesized compounds were fully determined by spectroscopic techniques.

**Scheme:**
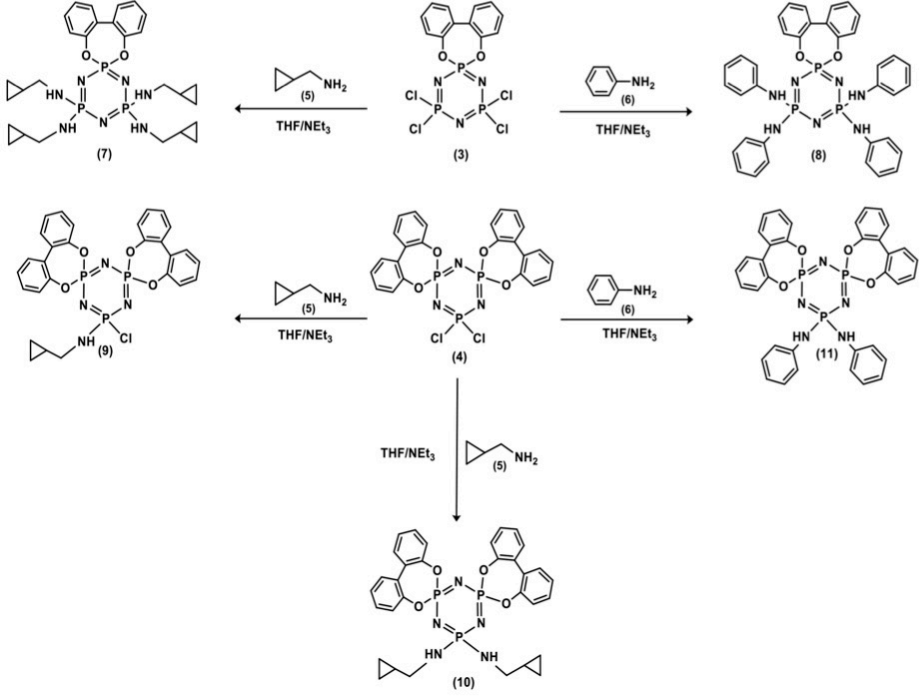
Synthesis of the new cyclotriphosphazene derivatives (7–11).

## 2. Experimental

### 2.1. Materials and methods

All reagents and solvents were purchased from commercial suppliers and used without further purification. Analytical thin layer chromatography (TLC) was performed on silica gel plates (Kieselgel 60 Å, 0.25 mm thickness; Merck, Darmstadt, Germany) with a F254 indicator. Column chromatography was performed on silica gel (Merck, Kieselgel 60 Å, 230–400 mesh). The melting point was measured with a Gallenkamp apparatus using a capillary tube. The elemental analyses were obtained using a Thermo Fisher Finnigan FlashEA 1112 instrument (Waltham, MA, USA). Mass analyses were recorded on a Bruker MALDI-TOF spectrometer (Billerica, MA, USA) using 1,8,9-anthrasenetriol (DIT) (for compounds 8, 9, and 11) and 2,5- dihydroxybenzoic acid (for compounds 7 and 10) as a matrix. NMR spectra ( ^1^ H and ^31^ P) were recorded for the newly synthesized compounds in CDCl _3_ solutions on a Varian INOVA 500 MHz spectrometer (Palo Alto, CA, USA). IR spectra were recorded between 4000 and 500 cm ^-1^ using a PerkinElmer Spectrum 100 FTIR spectrometer (Waltham, MA, USA) with an attenuated total reflection accessory featuring a zinc selenide crystal. All spectroscopic data are given in Supporting information.

### 2.2. X-ray data collection and structure refinement

Intensity data were recorded on a Bruker APEX II QUAZAR diffractometer. Absorption correction by multiscan was applied [26] and space groups were determined using XPREP implemented in APEX2 [27]. The molecular structures were determined using the direct methods procedure in SHELXS-97 and refined by full-matrix least squares on F2 using SHELXL-97 [28]. All nonhydrogen atoms were refined with anisotropic displacement factors and C-H hydrogen atoms were placed in calculated positions and allowed to ride on the parent atom. The final geometrical calculations and the molecular drawings were performed with the PLATON [29], MERCURY [30], and DIAMOND (Version 3.1) [31] programs. The molecular structure determination was deposited with the Cambridge Crystallographic Data Centre with reference CCDC 1941632 for compound 11.

### 2.3. Syntheses

The mono-spiro- (3), and bis-spiro- (4) 2,2′ -dioxybiphenyl-substituted cyclotriphosphazenes were synthesized from the reaction of hexachlorocyclotriphosphazene (N _3_ P _3_ Cl6) (1) with 2, 2′ -dioxybiphenyl (2), according to the literature [32].

#### 2.3.1. Synthesis of compound 7

A solution of the triethylamine (0.98 g, 9.8 mmol) in THF (10.0 mL) was added to a stirred solution of compound 3 (1.0 g, 2.2 mmol) in THF (15.0 mL) under an argon atmosphere in a 100-mL 3-necked roundbottomed flask. Next, cyclopropanemethylamine (5) (0.69 g, 9.8 mmol) in THF (10.0 mL) was added to the stirred solution. The reaction mixture was then refluxed for 3 days, followed by TLC on silica gel plates using THF and n-hexane (1:2) as the eluent. The absence of starting material and the formation of one product was observed. The triethylamine hydrochloride (NEt _3_ .HCl) and any other insoluble materials were filtered off. The solvent was removed under reduced pressure. The crude product was subjected to column chromatography using THF: n-hexane (1:2) as the mobile phase. The product (7), mono–spiro-(2,2′ -dioxybiphenyl)–tetra- (cyclopropanemethylamino)-cyclotriphosphazene (0.23 g, 18.0%, mp 166.0 °C), was obtained as white solid. Anal. Calc. for C _28_ H _40_ N _7_ O _2_ P _3_ ; C 56.09; H 6.72; N 16.35. Found; C 55.08; H 6.70; N 16.37%. MALDITOF- MS (Figure S1) (m/z): [M-H] ^+^ , 598.76 (calcd. 599.60). ^31^ P NMR (Figure S2) (500 MHz, CDCl _3_ ) δ (ppm); 27.13 (P(biph), t, ^2^ J _*PNP*_ = 62.94 Hz, 1P), 18.01 (P(NHR)2 , d, ^2^ J _*PNP*_ = 62.94 Hz, 2P). ^1^ H NMR (Figure S3) (500 MHz, CDCl _3_ , 298 K) δ (ppm); 7.46 (-O-C-C- *CH* ^4^ -, d, ^3^ J _*H3H4*_ = 8.09 Hz, 2H), 7.33 (-OC- C- *CH* ^2^ -, t, ^3^ J _*H2H3*_ = 7.39 Hz, 2H), 7.20(-O-C-C-C- *CH* ^3^ , t, ^3^ J _*H3H4*_ = 8.21, 2H,), 7.13(-O-C- *CH* ^1^ , d, ^3^ J _*H1H2*_ = 6.56Hz, 2H), 5.08 (-P-NH ^5^ -, s, 4H), 2.31 (-P-N-CH _6_^2^ -, m, 8H), 1.02 (P-N-C- *CH* ^7^ -, m, 4H), 0.44 (-C-C- *CH* ^8^ 8 _2_ -, d, ^3^ J _*H8H9*_ = 6.97, 8H) 0.15 (-C-C-C- *CH* ^9^_2_ -, d, ^3^ J _*H9H7*_ = 3.77, 8H). FT-IR (Figure S4) (ν : cm ^-1^ ) : 3214.8 (N-H); 3062.9 (C-H) _*aromatic*_ ; 2859.2 (C-H) _*aliphatic*_ ; 1500.2 (C=C) _*aromatic*_ ; 1221.3; and 1172.1 (P=N); 953.1 (P-O-C).

#### 2.3.2. Synthesis of compound 8

A solution of the triethylamine (0.6 g, 5.9 mmol) in THF (10.0 mL) was added to a stirred solution of compound 3 (0.6 g, 1.3 mmol) in THF (15.0 mL) under an argon atmosphere in a 100-mL three-necked round-bottomed flask. Next, aniline (5) (0.55 mg, 5.9 mmol) in THF (10.0 mL) was added to the stirred solution. The reaction mixture was then refluxed for 4 days, followed by TLC on silica gel plates using THF:n-hexane (1:2) as the eluent. The absence of starting material and the formation of one product was observed. The triethylamine hydrochloride (NEt _3_ .HCl) and any other insoluble materials were filtered off. The solvent was removed under reduced pressure. The crude product was subjected to column chromatography using THF and n-hexane (1:2) as the mobile phase. The product (8), mono–spiro-(2,2’-dioxybiphenyl)–tetra-(anilino)-cyclotriphosphazene (0.11 g, 12.0%), was obtained as oily. Anal. Calc. for C _36_ H _32_ N _7_ O _2_ P _3_ ; C 62.88; H 4.69; N 14.26. Found; C 62.85; H 4.65; N 14.20%. MALDI-TOF-MS (Figure S5) (m/z): [M+H] ^+^ , 689.07 (calcd. 687.62). ^31^ P NMR (Figures S6 and S7) (500 MHz, CDCl _3_ ) δ (ppm); 24.22 (P(biph), t, ^2^ J _*PNP*_ = 67.35 Hz, 1P), 5.73 (P(PhNH) _2_ , d, ^2^ J _*PNP*_ = 67.35 Hz, 2P). ^1^ H NMR (Figure S8) (500 MHz, CDCl _3_ , 298 K) δ (ppm); 7.55 (-O-C-C- *CH* ^4^ -, d, ^3^ J _*H3H4*_ = 8.0 Hz, 2H), 7.33 (-NHC-CH-CH- *CH* ^8^ -, m, 4H), 7.20 (-NH-C- *CH* ^6^ , m, 8H), 7.12 (-NH-C-CH- *CH* ^7^ , m, 8H), 6.9 (-O-C-CH-CH- *CH* ^3^ -, t, 3 JHH = 7.5 ^1^ Hz, 2H), 6.8 (-O-C-CH- *CH* ^2^ , t, 3 JHH = 7.58 Hz, 2H), 6.7 (-O-C- *CH* ^1^ , d, 3 JHH = 7.6 ^1^ Hz, 2H), 5.5 (-NH, broad, 4H). FT-IR (Figure S9) (ν : cm ^-1^ ) : 3226.3 (N-H); 3040.7 (C-H)aromatic ; 1601.6 and 1498.0 (C=C)aromatic ; 1221.9 and 1166.4 (P=N); 934.6 (P-O-C).

#### 2.3.3. Synthesis of compounds 9 and 10

A solution of the triethylamine (0.17 g, 1.74 mmol) in THF (10.0 mL) was added to a stirred solution of compound 4 (0.5 g, 0.87 mmol) in THF (10.0 mL) under an argon atmosphere in a 100-mL three-necked round-bottomed flask. Next, cyclopropanemethylamine (5) (0.12 g, 1.74 mmol) in THF (10.0 mL) was added to the stirred solution. The reaction mixture was then refluxed for 1 day, followed by TLC on silica gel plates using n-hexane: dichloromethane (1:2) as the eluent. The absence of starting material and the formation of two products was observed. The triethylamine hydrochloride (NEt _3_ .HCl) and any other insoluble materials were filtered off. The solvent was removed under reduced pressure. The crude product was subjected to column chromatography using n−hexane: DCM as the mobile phase. The first product (9), bis-spiro-(2,2′ - dioxybiphenyl)-mono-(cyclopropanemethylamino)-monochlorocyclotriphosphazene (0.159 g, 29.0%, mp 122.0 °C), was obtained as a white solid. Anal. Calc. for C _28_ H _24_ ClN _4_ O _4_ P _3_ ; C 55.23; H 3.95; N 9.20. Found; C 55.24; H 3.96; N 9.21%. MALDI-TOF-MS (Figure S10) (m/z): [M-H] ^+^ , 607.92 (calcd. 608.90). ^31^ P NMR (Figures S11 and S12) (500 MHz, CDCl _3_ ) δ (ppm); 29.40 (P(NHR)Cl, t, ^2^ J _*PNP*_ = 69.57 Hz, 1P), 23.01 (P(biph), d, ^2^ J _*PNP*_ = 69.57 Hz, 2P). ^1^ H NMR (Figure S13) (500 MHz, CDCl _3_ , 298 K) δ (ppm); 7.55 (-O-C-C- *CH* ^4^ -, d, ^3^ J _*H3H4*_ = 7.33 Hz, 4H), 7.44 (-O-C-C- *CH* ^2^ -,t, ^3^ J _*H2H3*_ = 7.33 Hz, 4H ), 7.39 (-O-C-CC- *CH* ^3^ , t, ^3^ J _*H3H4*_ = 8.00, 4H), 7.33 (-O-C- *CH* ^1^ , d, ^3^ J _*H1H2*_ = 7.56Hz, 4H), 3.76 (-P-NH5 -, s, 1H), 2.91 (-P-N- *CH* ^6^ 2 -, m, 2H), 0.90 (P-N-C- *CH* ^7^ -, m, 1H), 0.55 (-C-C- *CH* ^8^ 2 -, t, 3 JHH = 6.31, 2H) 0.23 (-C-C-C-CH 92 -, t, 3 JHH = 6.31, 2H). FT-IR (ν : cm ^-1^ ) : 3244.4 (N-H); 3065.1 (C-H)aromatic ; 2850.3 (C-H)aliphatic ; 1500.2 (C=C)aromatic ; 1224.7 and 1173.4 (P=N); 941.6 (P-O-C).

The second product (10), bis-spiro-(2,2′ -dioxybiphenyl)-di-(cyclopropanemethylamino) cyclotriphosphazene (0.10 g, 18.0%, mp 171.0 °C), was also obtained as a white solid. Anal. Calc. for C _28_ H _24_ ClN _4_ O _4_ P _3_ ; C 59.72; H 5.01; N 10.88 Found; C 59.69; H 4.98; N 10.72%. MALDI-TOF-MS (Figure S14) (m / z): [M+H] ^+^ , 643.61 (calcd. 643.56). ^31^ P NMR (Figures S15 and S16) (500 MHz, CDCl _3_ ) δ (ppm); 26.4 (P(biph), d, ^2^ J _*PNP*_ = 66.88 Hz, 2P), 18.45 (P(NHR) _2_ , t, ^2^ J _*PNP*_ = 66.88 Hz, 1P). ^1^ H NMR (Figure S17) (500 MHz, CDCl _3_ , 298 K) δ (ppm); 7.53 (-O-C-C- *CH* ^4^ -, d, ^3^ J _*H3H4*_ = 7.90 Hz, 4H), 7.42 (-O-C-C- *CH* ^2^ -,t, ^3^ J _*H2H3*_ = 7.76 Hz, 4H), 7.31(-O-C-C-C- *CH* ^3^ , t, ^3^ J _*H3H4*_ = 7.05, 4H), 7.28(-O-C- *CH* ^1^ , d, ^3^ J _*H1H2*_ = 1.86Hz, 4H), 3.76 (-P-NH5 -, s, 2H), 2.57 (-P-N- *CH* ^6^ 2 -, m 4H), 0.88 (P-N-C- *CH* ^7^ -, m, 2H), 0.55 (-C-C- *CH* ^8^ 2 -, t, 3 JHH = 6.71, 4H) 0.25 (-C-C-C-CH 92 -, t, ^3^ J _*H9H7*_ = 6.71, 4H). FT-IR (ν : cm ^-1^ ) : 3249.3 (N-H); 3062.9 (C-H)aromatic ; 2918.5 and 2870.3 (C-H)aliphatic ; 1500.4 (C=C)aromatic ; 1225.0 and 1160.0 (P=N); 1013.8 and 941.6 (P-O-C).

#### 2.3.4. Synthesis of compound 11

A solution of the triethylamine (0.35 g, 3.48 mmol) in THF (10.0 mL) was added to a stirred solution of compound 4 (0.5 g, 0.87 mmol) in THF (10.0 mL) under an argon atmosphere in a 100-mL three-necked round-bottomed flask. Next, aniline (0.32 g, 3.48 mmol) in THF (10.0 mL) was added to the stirred solution. The reaction mixture was then refluxed for 3 days, followed by TLC on silica gel plates using n-hexane and dichloromethane (1:2.5) as the eluent. The triethylamine hydrochloride (NEt _3_ .HCl) and any other insoluble materials were filtered off. The solvent was removed under reduced pressure. The crude product was chromatographed on silica gel using DCM and n-hexane (2.5:1) solvent system as the mobile phase. The product (11), bis-spiro-(2,2′ -dioxybiphenyl)–bis-(anilino)-cyclotriphosphazene (0.11 g, 19.0%, mp 162.7 °C), was obtained as a white solid. This compound was recrystallized from DCM and n-hexane (1:1) and was acquired as colorless block crystals that were suitable for X-ray crystallography. Anal. Calc. for C _36_ H _28_ N _5_ O _4_ P _3_ ; C 62.89;H 4.10; N 10.19. Found; C 62.87; H 4.09; N 10.20%. MALDI-TOF-MS (m/z): [M] ^+^ , 687.69 (calcd. 687.57) (Figure S18). ^31^ P NMR (Figures S19 and S20) (500 MHz, CDCl _3_ ) δ (ppm); 25.42 (P(biph), d, ^2^ J _*PNP*_ = 72.45 Hz, 2P), 6.34 (P(PhNH) _2_ , t, ^2^ J _*PNP*_ = 72.45 Hz, 1P). ^1^ H NMR (500 MHz, CDCl _3_ , 298 K) δ (ppm); 7.55 (-O-C-C- *CH* ^4^ -, d, ^3^ J _*H3H4*_ = 8. ^1^ Hz, 4H), 7.33 (-NHC-CH-CH- *CH* ^8^ -, m, 2H), 7.20 (-NH-C- *CH* ^6^ , m, 4H), 7.12 (-NH-C-CH- *CH* ^7^ , m, 4H), 6.9 (-O-C-CH-CH- *CH* ^3^ -, t, 3 JHH = 7.6 ^1^ Hz, 4H), 6.8 (-O-C-CH- *CH* ^2^ , t, 3 JHH = 7.58 Hz, 4H), 6.7 (-O-C- *CH* ^1^ , d, 3 JHH = 7.6 ^1^ Hz, 4H), 5.5 (-NH, broad, 2H). FT-IR (ν : cm ^-1^ ) : 3262.4 (N-H); 3040.1 (C-H)aromatic ; 1717.2 (C=C)aromatic ; 1261.7 and 1095.9 (P=N); 1018.3 and 1043.5 (P-O-C).

## 3. Results and discussion

### 3.1. Syntheses of the cyclotriphosphazene derivatives

**Figure 1:**
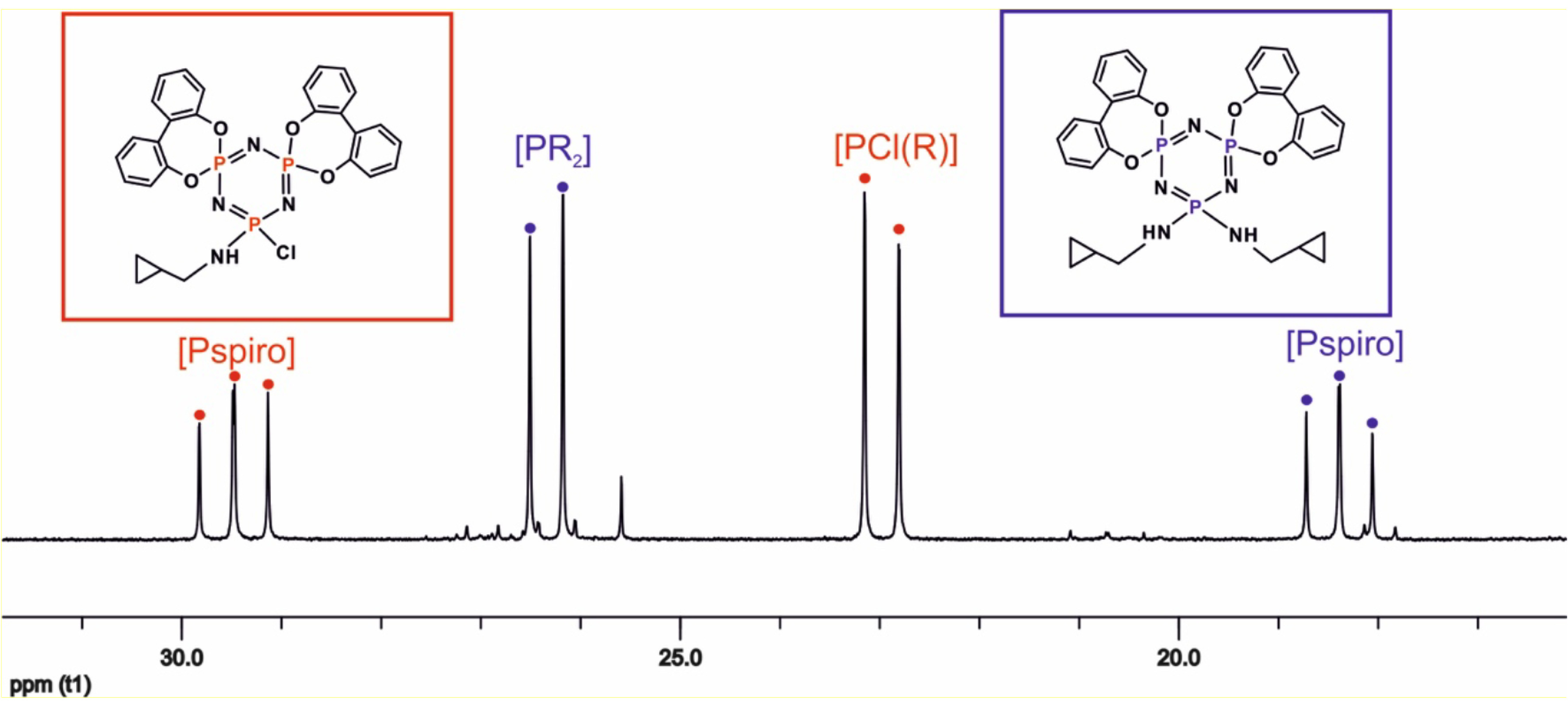
Proton decoupled ^31^ P NMR spectrum of the reaction mixture of compounds 9 and 10.

The cyclopropanemethylamino- and anilino-substituted spiro-2,2′ -dioxybiphenyl cyclo-triphosphazene derivatives (7–11) were synthesized as shown in Scheme. Synthesis of the new cyclotriphosphazene derivatives started with preparing the mono- and dispiro-2,2′ -dioxybiphenyl-substituted cyclotriphosphazenes (3 and 4). The hexachlorocyclotriphosphazene (N _3_ P _3_ Cl _6_ ) (1) was reacted with 2,2′ -dioxybiphenyl (2) at a 1:2 molar ratio in the presence of K _2_ CO _3_ in acetone solution at room temperature and spiro-2,2′ -dioxybiphenyl cyclotriphosphazene derivatives (3 and 4) were obtained [32]. The nucleophilic substitution reaction of monospiro- 2,2′ -dioxybiphenyl cyclotriphosphazene (3) with cyclopropanemethylamine (5) and aniline (6) in the presence of Et3 N in THF at reflux, yielded monospiro-(2,2’-dioxybiphenyl)–tetra-(cyclopropanemethylamino)- cyclotriphosphazene (7) and monospiro-(2,2’-dioxybiphenyl)–tetra-(anilino)-cyclotriphosphazene (8), respectively. The reaction of bis-spiro-2,2′ -dioxybiphenyl cyclotriphosphazene (4) with cyclopropanemethylamine (5) resulted in bis-spiro-(2,2′ -dioxybiphenyl)–mono-(cyclopropanemethylamino)–monochloro-cyclotriphosphazene (9) and bis-spiro-(2,2′ -dioxybiphenyl)–bis-(cyclopropanemethylamino) cyclotriphosphazene (10). The formed compounds (9 and 10) in the reaction mixture were checked by TLC and proton decoupled ^31^ P NMR spectroscopy (Figure 1). The pure compounds (9 and 10) were obtained in moderate yields after column chromatography. On the other hand, only one product (compound 11) was obtained from the reaction of compound 4 with aniline (6) under the same reaction conditions. All of the novel cyclotriphosphazene derivatives (7–11) were isolated by column chromatography using silica gel. The cyclopropanemethylamino- and anilino-substituted spiro-2,2′ -dioxybiphenyl cyclotriphosphazene derivatives (7–11) were soluble in known organic solvents, such as tetrahydrofuran, dichloromethane, and chloroform.

### 3.2. Characterizations of the cyclotriphosphazene derivatives (7–11)

**Figure 2:**
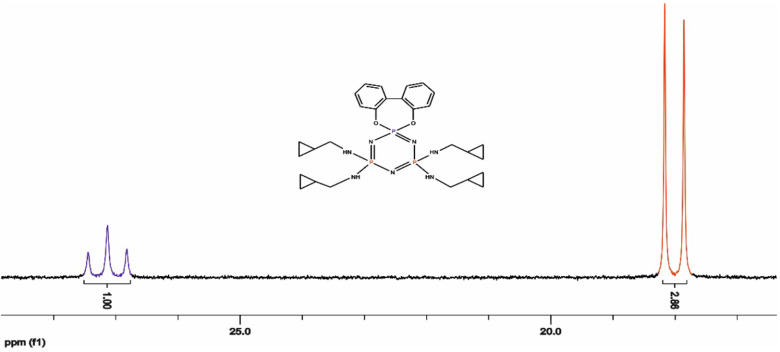
^31^ P decoupled NMR spectrum of compound 7.

The cyclopropanemethylamino- and anilino-substituted spiro-2,2′ -dioxybiphenyl cyclotriphosphazene derivatives (7–11) were fully characterized by elemental analysis, MALDI-TOF MS, FT-IR, ^1^ H and ^31^ P NMR spectroscopies (Supporting information). All spectroscopic data were in good agreement with the proposed structures, as shown in Scheme. The proton decoupled ^31^ P NMR spectrum of monospiro-(2,2’-dioxybiphenyl)– tetra-(cyclopropanemethylamino) cyclotriphosphazene (7) is depicted in Figure 2. The proton decoupled ^31^ P NMR spectra of the monospiro-2,2′ -dioxybiphenyl tetra–amino-substituted cyclotriphosphazenes (7 and 8) were observed as AX2 spin systems owing to two different phosphorus nuclei of the cyclotriphosphazene ring. As expected, the chemical shifts of the spiro groups [P(biph)] were observed between 27.0 and 24.0 ppm [32]. The chemical shifts of the PR2 groups depended on the chemical structure of the R group. The P(NHR) _2_ groups for compound 7 had a chemical shift at 18.1 ppm, whereas the chemical shift of the [P(PhNH) _2_ ] groups for compound 8 was observed at 5.7 ppm (Table 1). Moreover, the MALDI-TOF MS and elemental analyses of the cyclotriphosphazene derivatives (7 and 8) revealed that four chlorine atoms were substituted by the cyclopropanemethylamino or aniline groups. The measured MALDI-TOF MS spectra of compounds 7 and 8 showed molecular ion peaks at m/z 599.25 and 689.07, respectively (Figures S1 and S5).

**Table 1:** ^31^ P NMR parameters of the cyclotriphosphazene derivatives (7–11).

Compound	Spin system	2J(PNP) [Hz]						
		P(biph) (1)	P(NHR)Cl (2)	P(NHR) _2_ (3)	P(PhNH) _2_ (4)	1,2	1,3	1,4
7	AX2	27.13	-	18.01	-	-	62.94	-
8	AX2	24.22	-		5.73	-	-	67.35
9	AX2	23.01	29.40	-	-	69.57	-	-
10	A2X	26.41	-	18.45	-	-	66.88	-
11	A2X	25.42	-	-	6.34	-	-	72.45

a 202.38 MHz 31P NMR chemical shifts (ppm) in CDCl _3_ solution.

Although the cyclopropanemethylamino-substituted cyclotriphosphazene derivatives (9 and 10) had two different phosphorus environments within the molecule, the ^31^ P NMR spectrum of compound 9 exhibited an AX2 spin system, whereas compound 10 exhibited an A2 X spin system. The signals of compound 9 consisted of a triplet for the P(NHR)Cl group at 29.4 ppm and a doublet for the spiro [P(biph)] groups at 23.0 ppm ( ^2^ J _*PNP*_ = 69.6 Hz) (Figure S11). On the other hand, the [P(biph)] groups were observed as a doublet at 26.4 ppm and the P(NHR) _2_ group appeared as a triplet at 18.45 ppm in the ^31^ P NMR spectrum of compound 10 (Figure S15). Further structural verification was obtained via the MALDI-TOF MS spectra. The [M-H] ^+^ and [M]+ ions peaks of compounds 9 and 10 were marked as 607.92 Da and 643.61 Da, respectively (Figures S10 and S14). The proton decoupled ^31^ P NMR spectrum of the bis-spiro-(2,2′ -dioxybiphenyl)–bis-(anilino)-cyclotriphosphazene derivative (11) was observed as an A2 X spin system, owing to 2 different phosphorus environments within the molecules, as was expected. The signals consisted of a doublet (at ca. δ = 25.4 ppm) for the spiro [P(biph)] groups and a triplet (at ca. δ = 6.3 ppm) for the P(PhNH) _2_ group. The elemental analysis and MALDI-TOF MS data of compound 11 confirmed that two chlorine atoms on the cyclotriphosphazene ring were substituted by the anilino group. The ^1^ H NMR spectra of the cyclopropanemethylamino-substituted spiro-2,2′ -dioxybiphenyl cyclotriphosphazene derivatives (7, 9, and 10) showed similarity (Figures S3, S13, and S17). The aromatic protons were observed at δ = 7.6–7.1 ppm, and the aliphatic protons were observed at 2.6–0.1 ppm in the ^1^ H NMR spectra. The -NH protons of compounds 9 and 10 appeared at about 3.5 ppm, whereas the -NH protons of compound 7 were observed at 5.1 ppm. The aromatic protons of the anilino-substituted spiro-2,2′ -dioxybiphenyl cyclotriphosphazene derivatives (8 and 11) appeared between 7.6 and 6.5 ppm. The -NH protons of the cyclotriphosphazene derivatives (8 and 11) were observed at 5.5 ppm.

FT-IR spectra of cyclotriphosphazene derivatives (7–11) exhibited characteristic stretching bands for P=N- between 1221 and 1096 cm ^-1^ . The vibration bands assignable to the stretching of the N-H were observed at 3260–3220 cm ^-1^ . While the stretching peaks corresponding to aromatic C-H were marked between 3260 and 3220 cm ^-1^ , the stretching bands of aliphatic C-H were seen at about 2850 cm ^-1^ . The vibration bands of aromatic -C=C- appeared at approximately 1500 cm ^-1^ and peaks of P-O-C were observed between 1013 and 930 cm ^-1^ .

#### 3.2.1. Crystal structure description of compound 11

**Figure 3:**
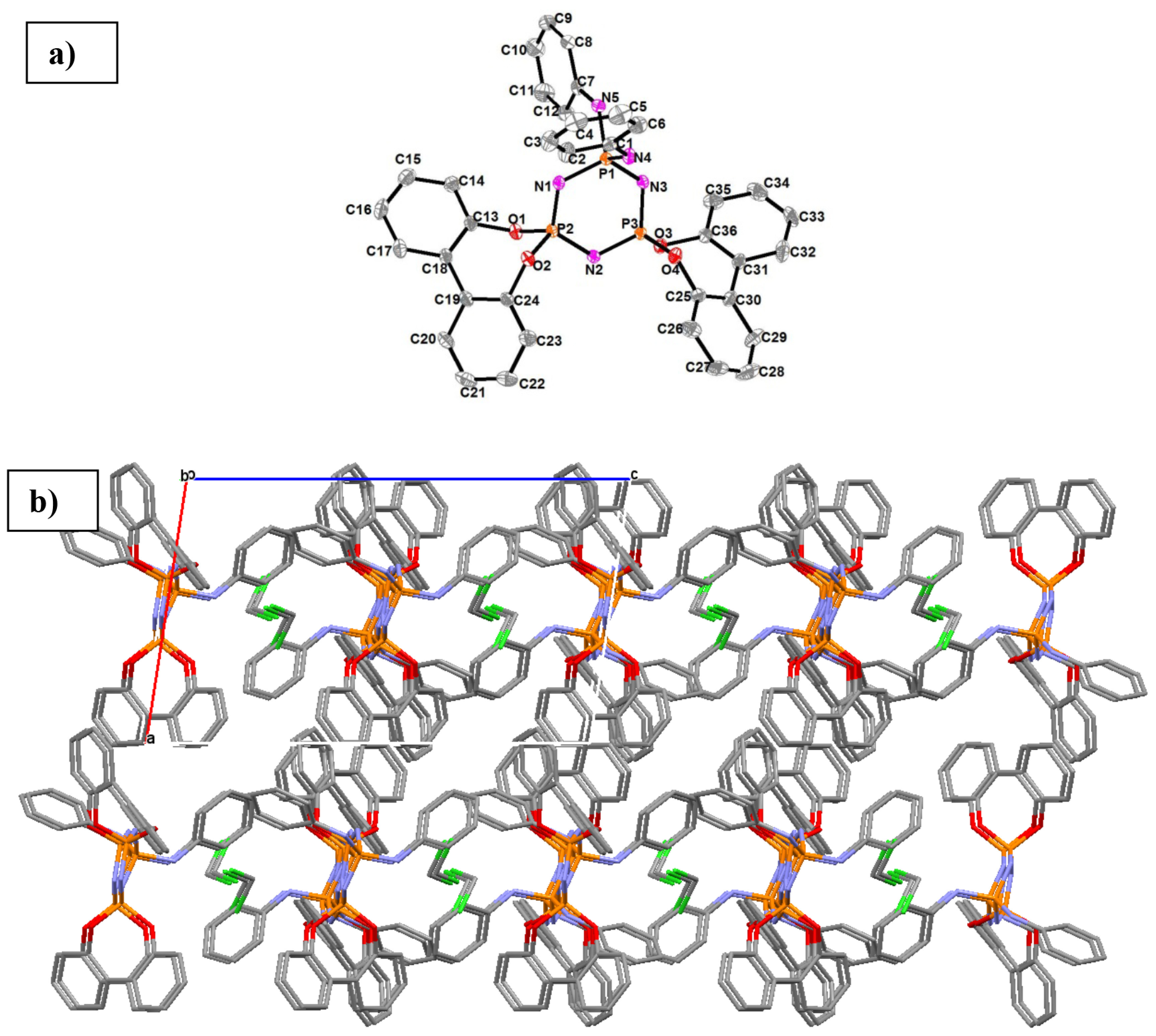
a. Crystal structure of compound 11 showing the atom numbering scheme. Displacement ellipsoids are drawn at the 30% probability level. The hydrogen atoms and dichloromethane solvent molecule were omitted for clarity. b) View of the 2 ×2 ×2 crystal packing of compound 11 along the b axis.

The crystal structure of compound 11 was determined by single-crystal X-ray crystallography. The molecular structure is shown in Figure 3a and the selected data collection and refinement details are presented in Table 2. Compound 11 crystallized in the monoclinic crystal system P21/c space group. The unit cell contained a dichloromethane solvent molecule per molecule (Z = 4). The view of the 2 ×2 ×2 crystal packing of compound 11 along the b axis is given in Figure 3b.

**Table 2:** X-ray crystallographic data and refinement parameters for compound 11.

Empirical formula	C36H28N5O4P3.CH2Cl2
Fw	772.47
T (K)	296 (2)
Crystal system	Monoclinic
Space group	P21/c
a (Å)	11.1447 (5)
b (Å)	17.7235 (8)
c (Å)	18.1830 (8)
β (°)	98.557 (2)
V (Å3)	3551.6 (3)
ρ (calcd.) (g cm ^-1^ )	1.445
Z	4
F (000)	1592
μ(mm−1) (MoKα)	0.367
Crystal size (mm3)	0.156 ×0.190 ×0.477
Reflection collected	51402
Independent reflection	6246
Data/restraints/parameters	6246 / 2 / 468
θ max (°)	25
h/k/l max	13 / 21 / 21
Tmin/Tmax	0.8440 / 0.945
Goodness-of-fit on F2	1.063
R [F2>2σ(F2)]	0.0467
wR [all reflections]	0.1293
Largest difference peak and hole (eÅ−3)	0.707 and –0.604

The 6-membered cyclotriphosphazene ring had a quite flattened chair conformation; the maximum deviation from planarity was seen on the N3 atom [0.181(2) Å]. The bond and conformational parameters of compound 11 are given in Table S1. The substituent effect on the bond parameters of the P3 N3 ring was observed; the P-N bond lengths involving the P1 phosphorus atom germinal were substituted with anilino units, whereas the P-N bond lengths involving the P2 and P3 phosphorus atoms spiro were substituted with 2,2′ -dioxybiphenyl groups. Similarly, The N-P-N bond angle was smaller for the P1 phosphorus atoms than those observed for the P2 and P3 atoms. The angles between the planes phenyl rings of the substituted groups were 39.85°and 42.92°, and the related C-C-C-C torsion angles were 43.0 (4)°and –41.2 (4)°(Table S1).

### 3.3. Chlorine replacement pattern

In the current work, the outcome of the ^31^ P NMR spectra and X-ray crystallography (for compound 11) of the new cyclophosphazene derivatives, including the 2,2′ -dioxybiphenyl moieties and amine groups (7–11), showed that compound 9 might have been formed via the SN2 pathway. The other compounds, 7, 8, 10, and 11, could be formed via both the SN2 and a proton abstraction-chloride elimination mechanism, respectively [10,11,13,15]. Hence, a triethylamine abstracted a proton from the PCl(NHR) (R = C6 H5 -, C4 H7 -) center in the cyclotriphosphazene ring, which was pursued by the loss of chloride ions, causing the formation of a three-coordinate phosphoranimine intermediate. Attack by another mole of amine derivatives (5 or 6) on this phosphoranimine led to the exhibited geminally-substituted product. Similar results were acquired with primary amines, such as 1,4-benzodioxan-6-amine, p-toluidine, and t -butylamine [10,11,13,15].

## 4. Conclusion

In this work, synthesis of the cyclopropanemethylamino- and anilino-substituted spiro 2,2′ -dioxybiphenyl cyclotriphosphazene derivatives (7–11) were described. The substitution of the chlorides was generally easy to control, and the target compounds were isolated in moderate yields by simple column chromatography. The molecular structures of the novel cyclotriphosphazene derivatives were characterized by various techniques, such as elemental analysis, MS, FT-IR, and ^1^ H and ^31^ P NMR spectroscopy. The molecular structure of compound 11 was also determined by single-crystal X-ray crystallography. Compound 1 ^1^ Had the P21/c space group and the monoclinic crystal system. The cyclopropanemethylamino- and anilino-substituted spiro-2,2′ -dioxybiphenyl cyclotriphosphazene derivatives may be offered as a small molecule model system for the design of new coordination polymers. We envisage that this molecule concept will be useful for optical materials and chemical sensors.

Supplementary Materials
