# Supplementary material for: Nucleophilic substitution reactions of monofunctional nucleophilic reagents with cyclotriphosphazenes containing 2,2-dioxybiphenyl units
Source: Turk J Chem. 2020 Feb 11;44(1):87–98. doi: 10.3906/kim-1907-45 (PMC7751811; doi:10.3906/kim-1907-45)
Supplement: Supplementary file 1 — Supplementary Materials [file turkjchem-44-87-sup001.pdf]

SUPPORTING INFORMATION

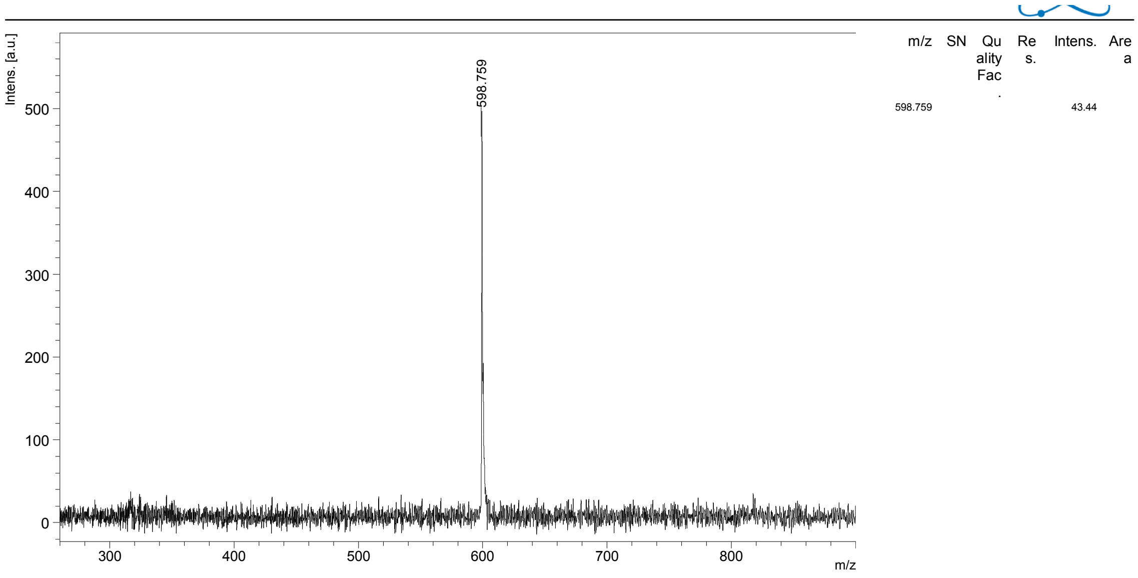

Figure S1. Mass spectrum of compound **7**

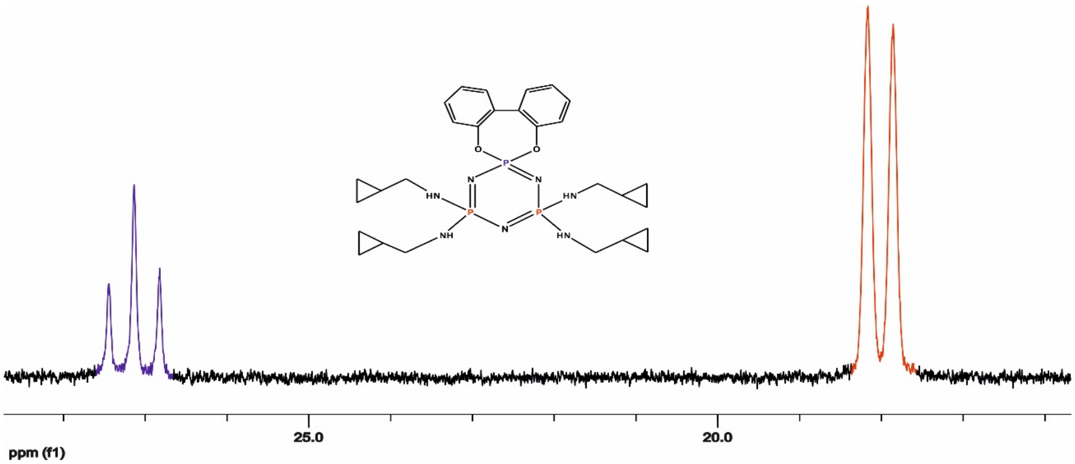

Figure S2:^{31}P coupled NMR spectrum of **7**

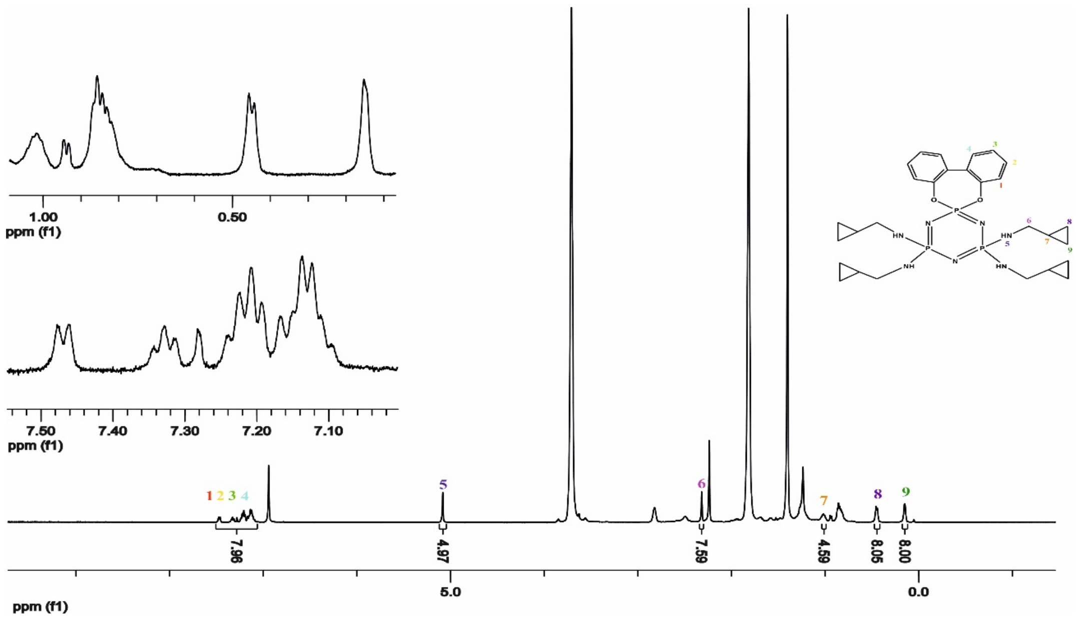

Figure S3: ^{1}H NMR spectrum of **7**

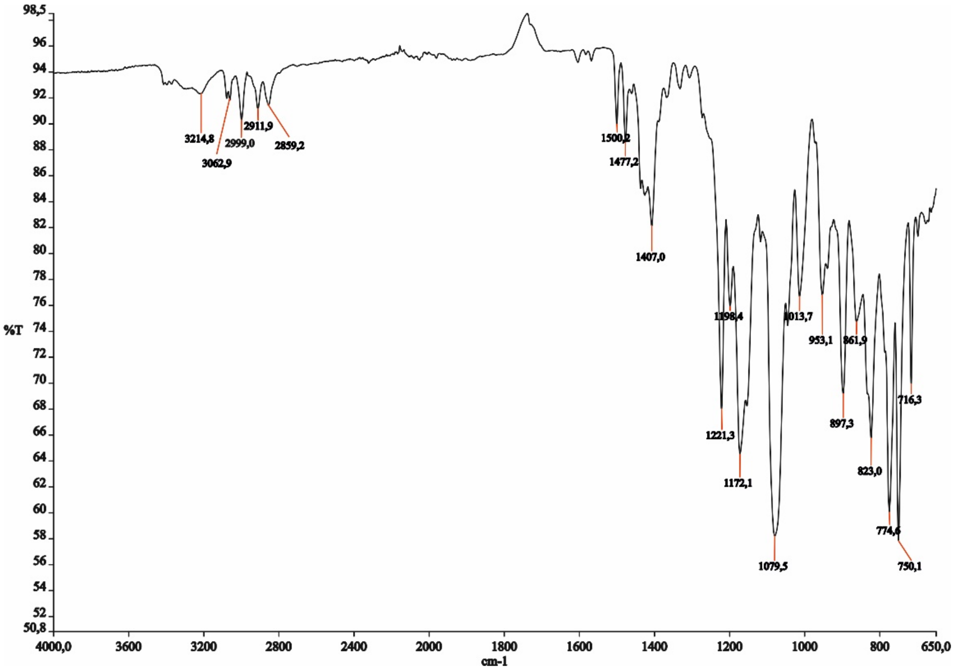

Figure S4: FT-IR spectrum of **7**

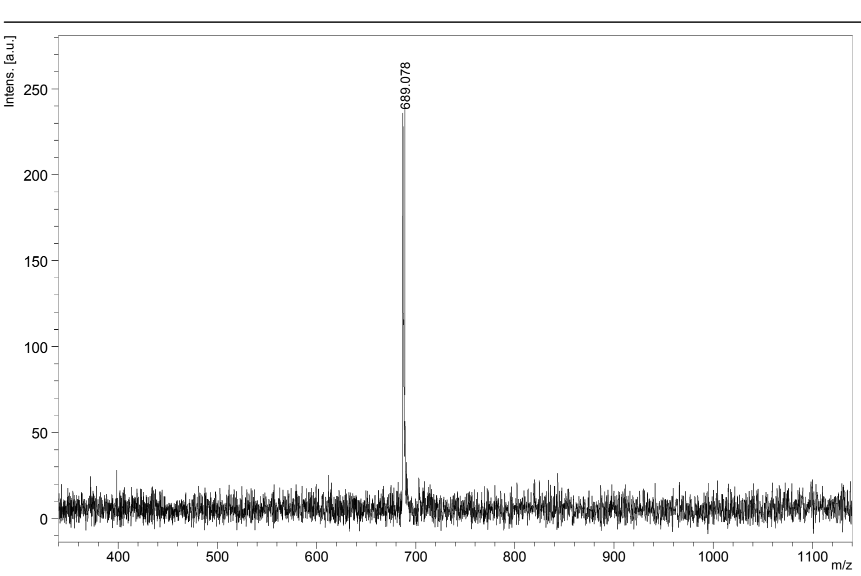

m/z	SN	Quality Fac.	Res.	Intens.	Area
689.078				51.07	

Figure S5. Mass spectrum of compound **8**

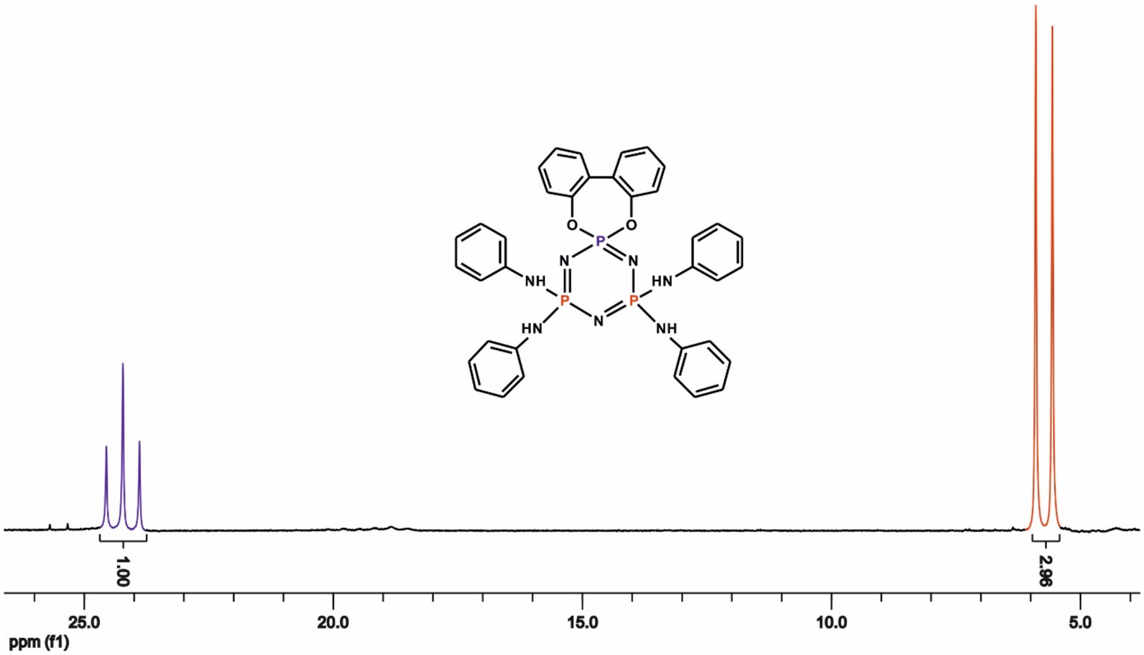

Figure S6:^{31}P decoupled NMR spectrum of **8**

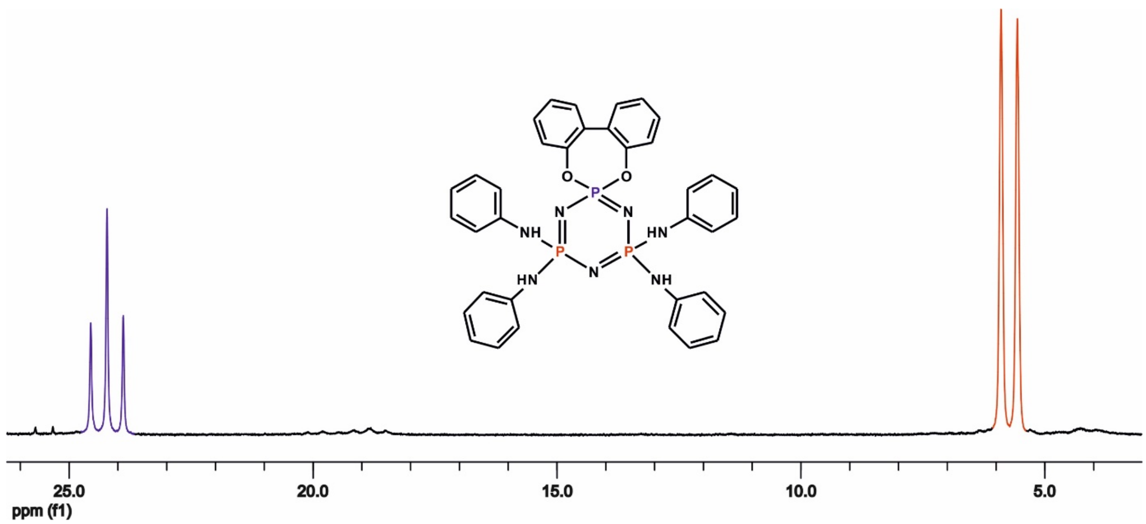

Figure S7:^{31}P coupled NMR spectrum of **8**

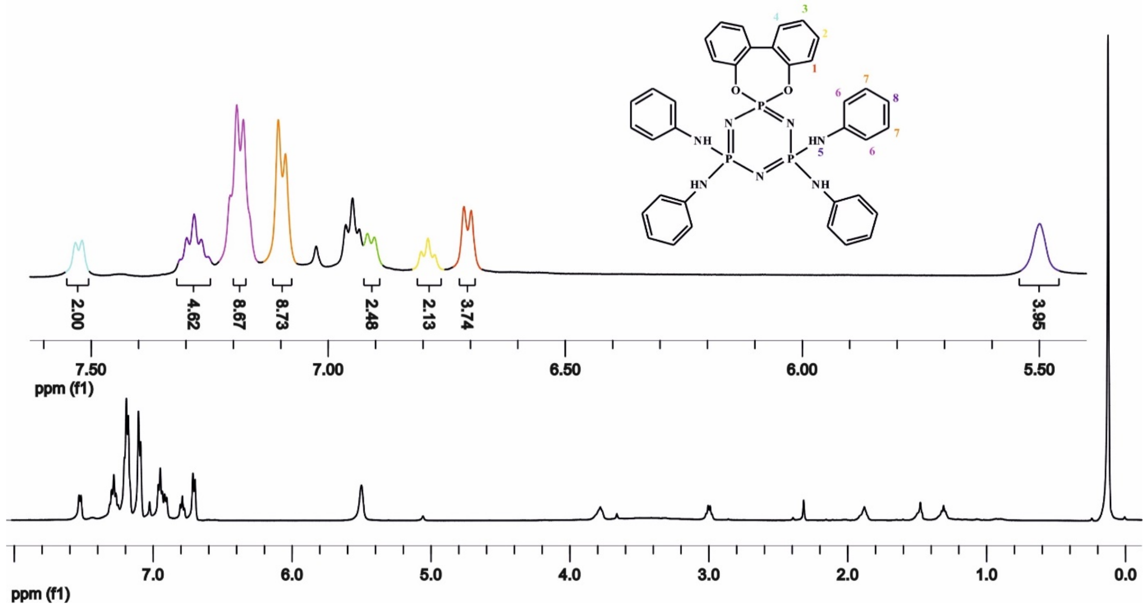

Figure S8:^{1}H NMR spectrum of **8**

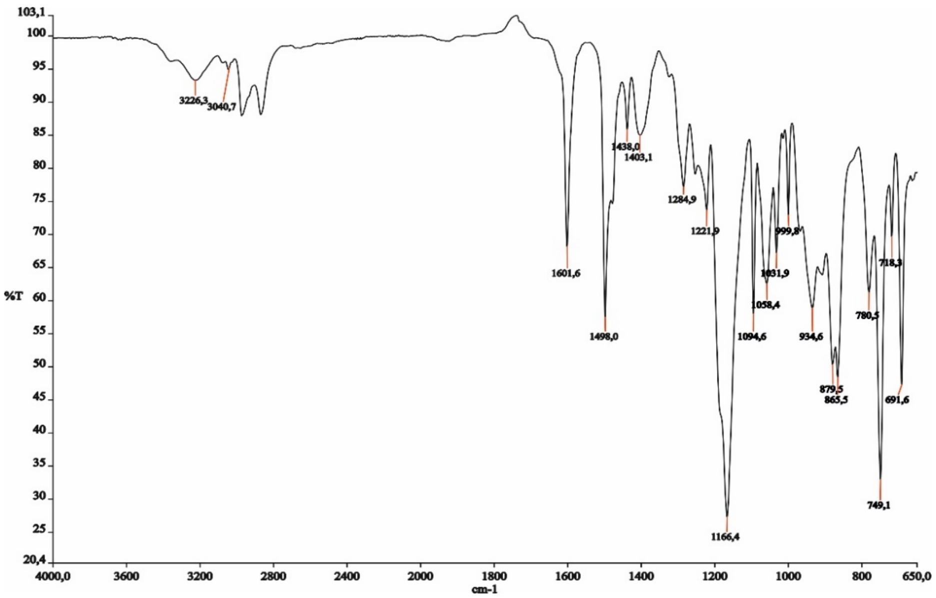

Figure S9: FT-IR spectrum of **8**

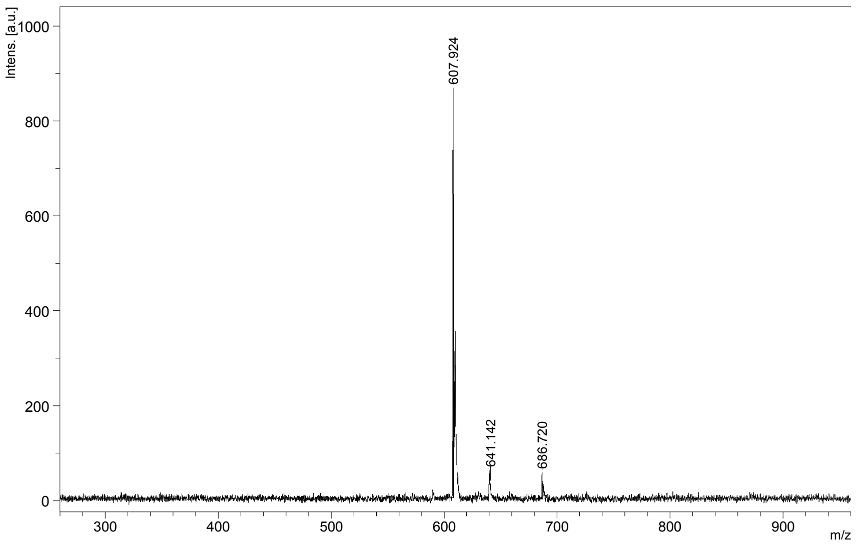

m/z	SN	Quality Fac.	Res.	Intens.	Area
607.924	70.5		1164	870.00	537
641.142				22.00	
686.720	4.7		1681	59.00	31

Figure S10. Mass spectrum of compound **9**

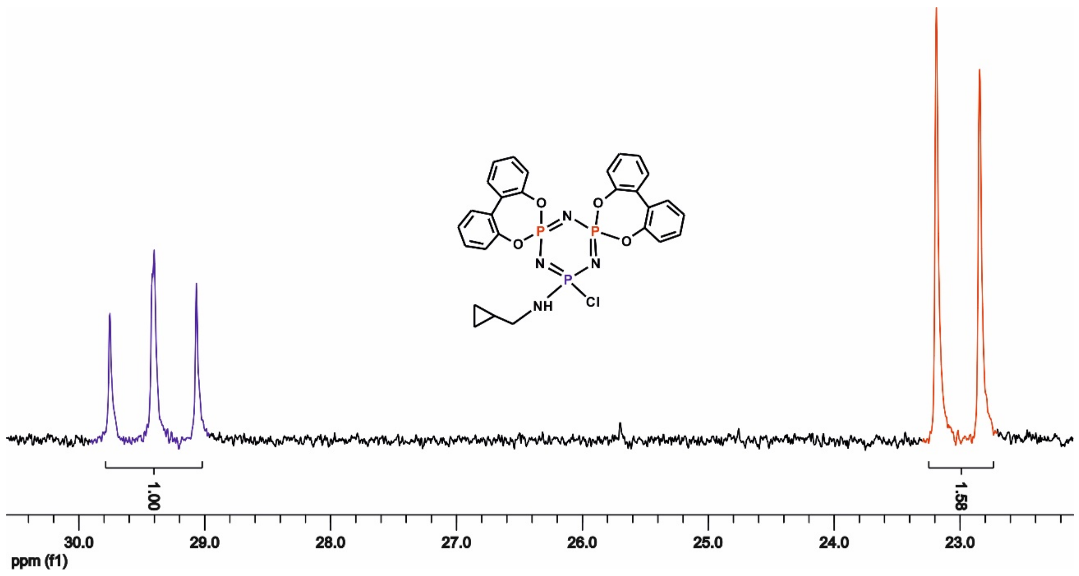

Figure S11: ^{31}P decoupled NMR spectrum of **9**

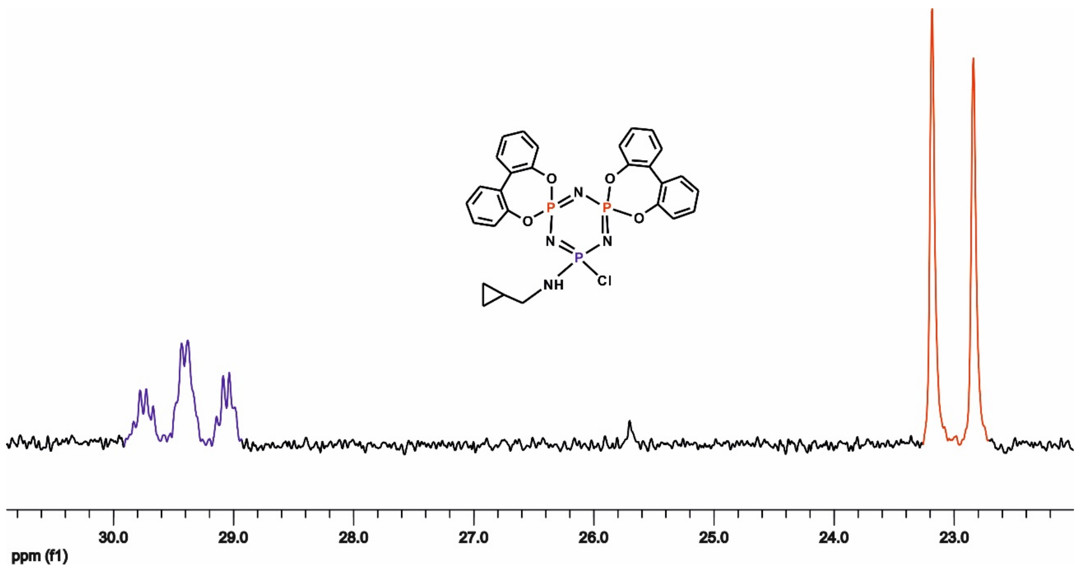

Figure S12:^{31}P coupled NMR spectrum of **9**

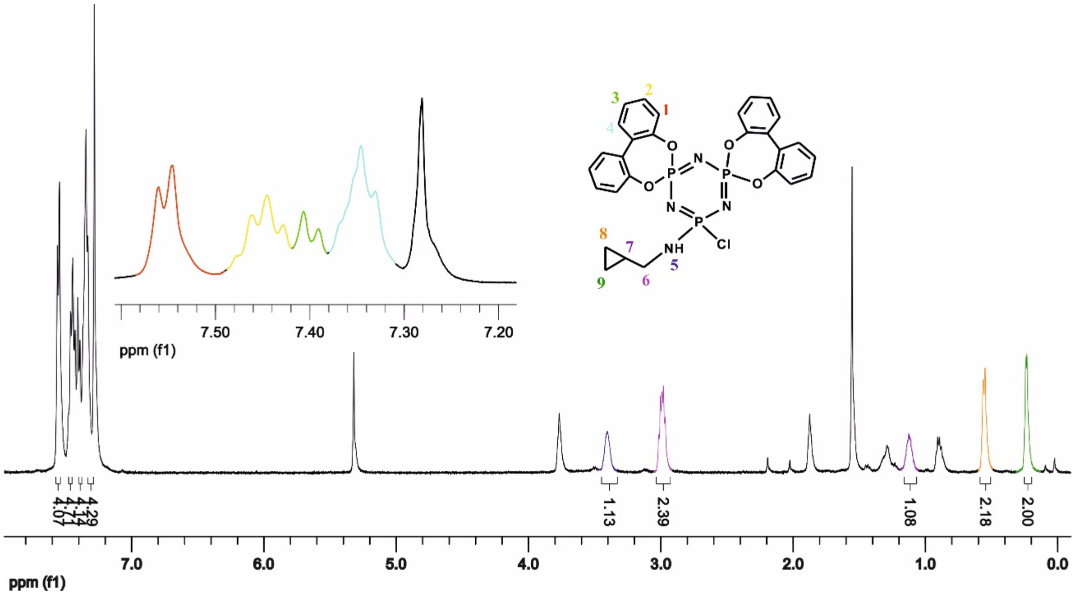

Figure S13:^{1}H NMR spectrum of **9**

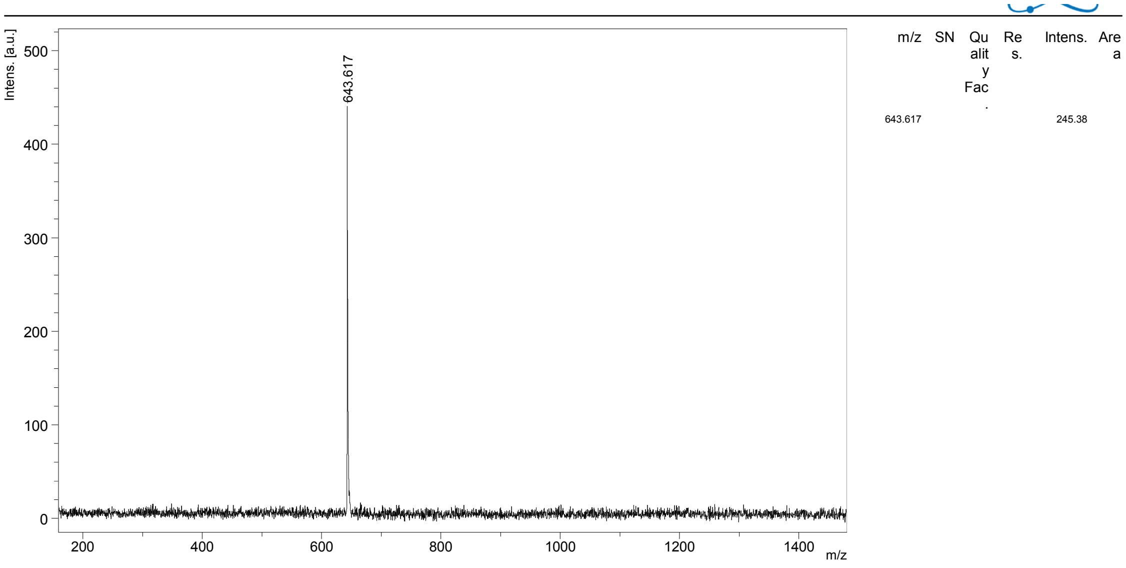

Figure S14. Mass spectrum of compound **10**

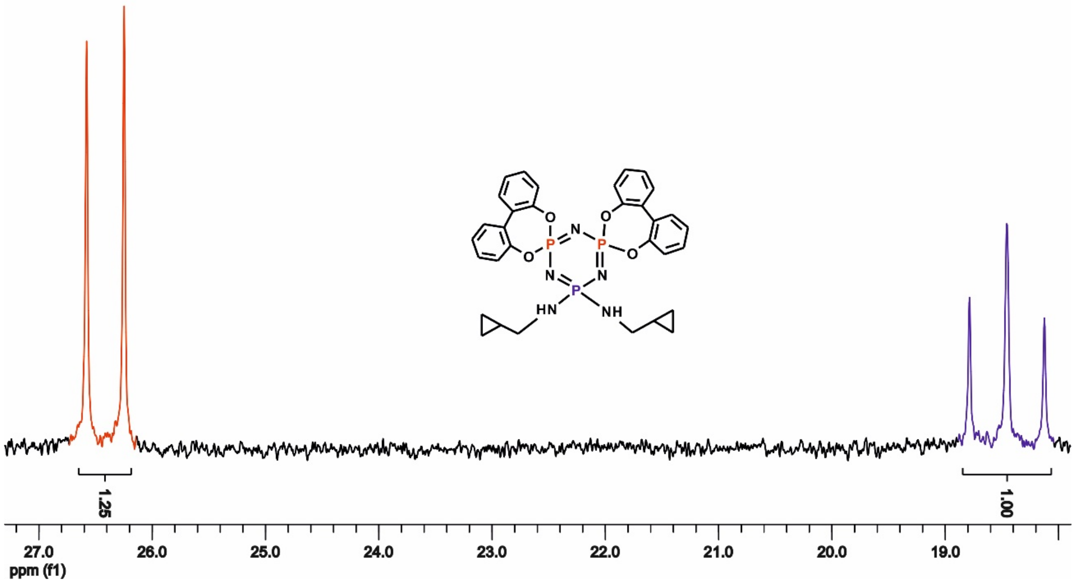

Figure S15. ^{31}P decoupled NMR spectrum of **10**

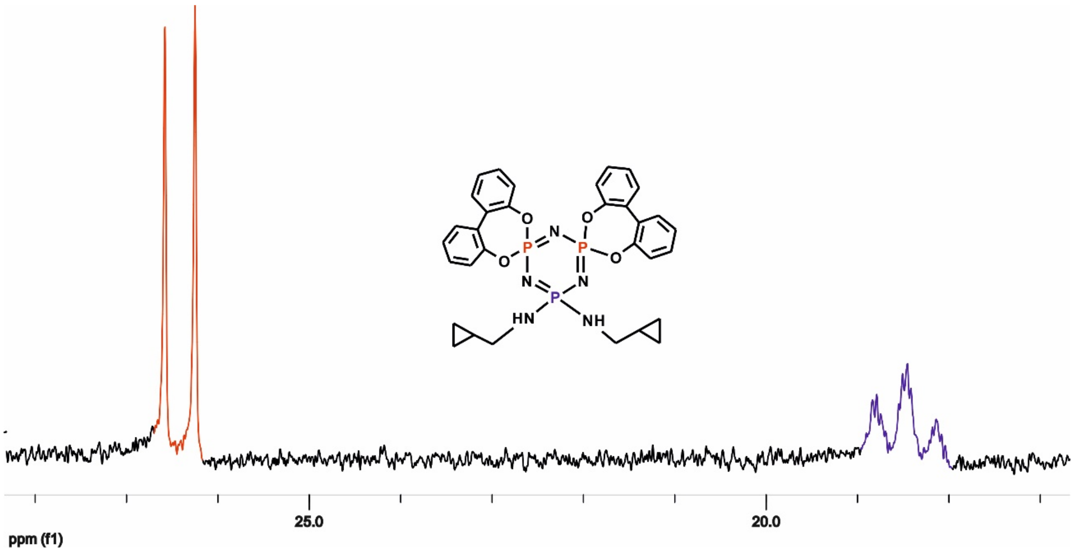

Figure S16. ^{31}P coupled NMR spectrum of **10**

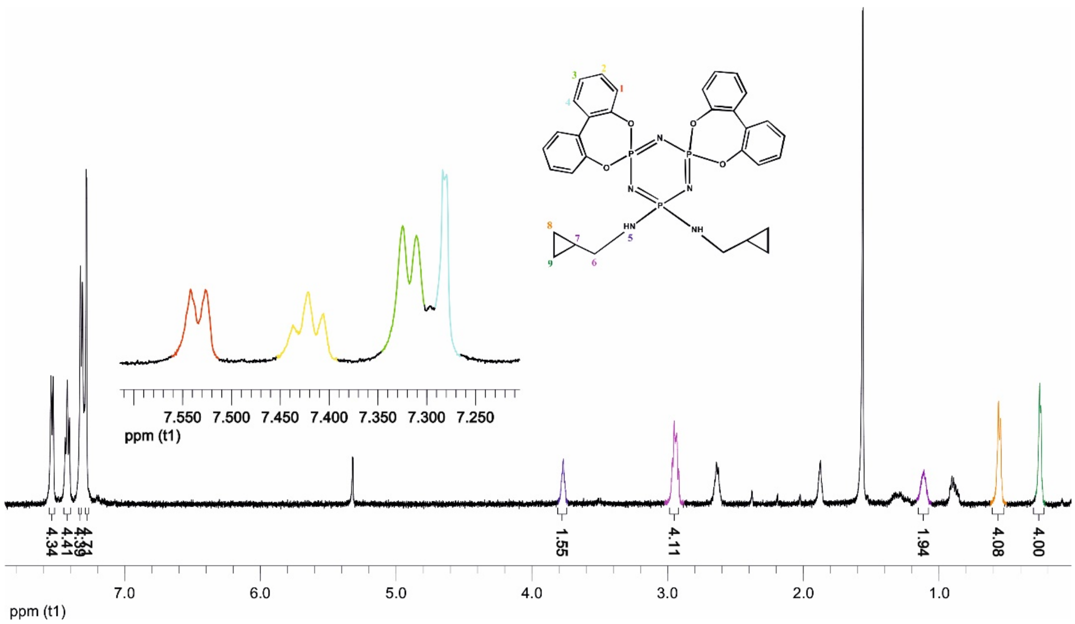

Figure S17: ^{1}H NMR spectrum of **10**

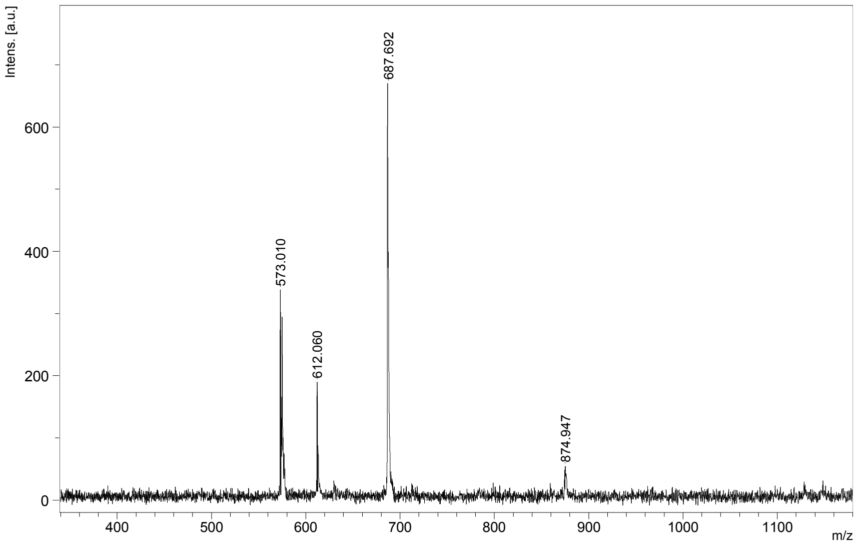

m/z	SN	Quality Fac.	Res.	Intens.	Area
573.010	21.9		942	337.52	238
612.060	12.4		908	188.92	159
687.692				398.65	
874.947	3.4		3635	53.62	18

Figure S18. Mass spectrum of compound **11**

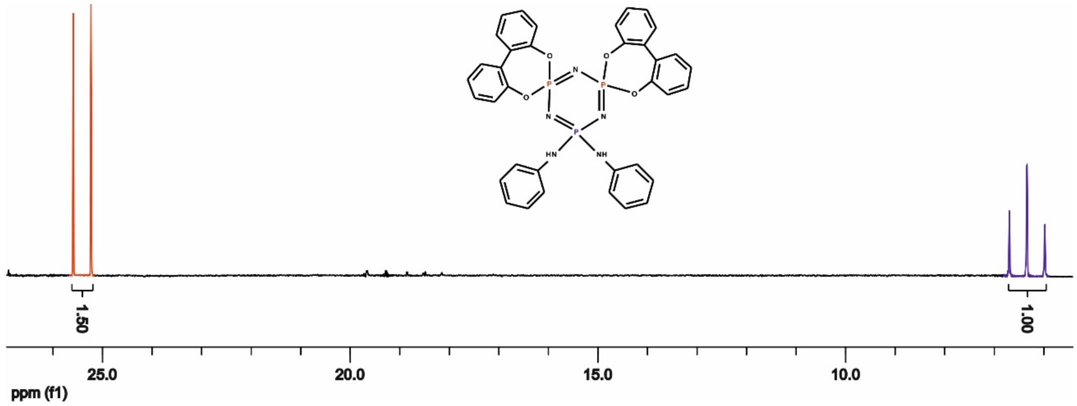

Figure S19. ^{31}P decoupled NMR spectrum of **11**

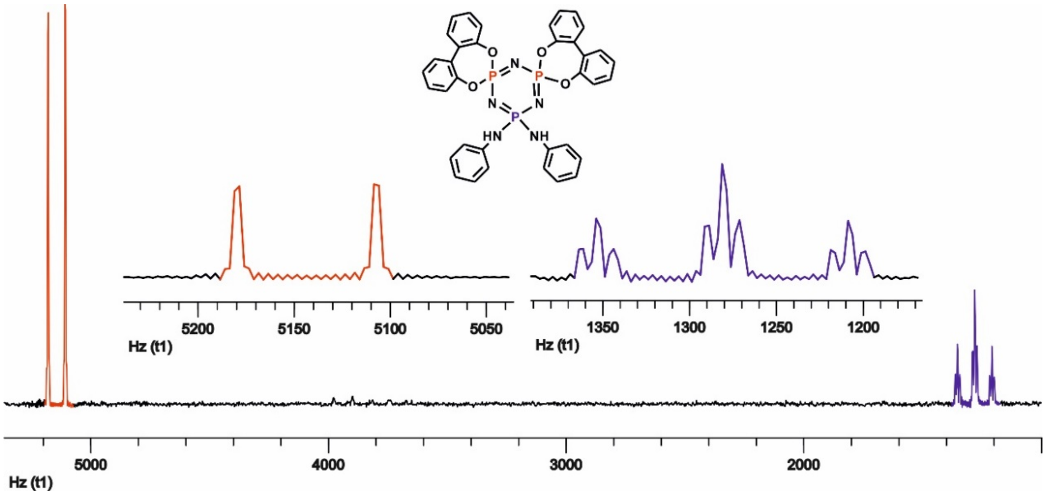

Figure S20. ^{31}P coupled NMR spectrum of **11**

Table S1. The selected bond and conformational parameters of compound **11**

**Bond lengths (Å)**	
**P1-N1**	1.594(2)
**P1-N3**	1.604(2)
**P2-N1**	1.569(2)
**P2-N2**	1.577(2)
**P3-N2**	1.579(2)
**P3-N3**	1.577(2)
**P1-N4**	1.647(3)
**P1-N5**	1.640(2)
**P2-O1**	1.596(2)
**P2-O2**	1.597(2)
**P3-O3**	1.586(2)
**P3-O4**	1.592(2)
**Bond Angles(°)**	
**N1-P1-N3**	115.02(12)
**N1-P2-N2**	118.91(12)
**N2-P3-N3**	117.80(12)
**P1-N1-P2**	121.99(14)
**P2-N2-P3**	120.35(14)
**P1-N3-P3**	119.28(14)
**N5-P1-N4**	102.85(13)
**O1-P2-O2**	101.87(10)
**O3-P3-O4**	102.80(10)
**Torsion Angles(°)**	
**C13-C18-C19-C24**	43.0(4)
**C25-C30-C31-C36**	-41.2(4)
**Conformation parameters of cyclotriphosphazene ring**	
**Max. deviation (Å)**	0.181(2) [on N3 atom]
**Puckering Amplitude Q (Å)**	0.2565(18)
θ **(°)**	122.5(4)
φ **(°)**	124.7(5)
